# Response to inhaled ceftazidime in patients with non‐cystic fibrosis bronchiectasis and chronic bronchial infection unrelated to *Pseudomonas aeruginosa*

**DOI:** 10.1111/crj.13534

**Published:** 2022-08-26

**Authors:** Vanessa Riveiro, Ana Casal, José M. Álvarez‐Dobaño, Tamara Lourido, Pedro Suárez‐Artime, Carlota Rodríguez‐García, Lucía Ferreiro, María E. Toubes, Luis Valdés

**Affiliations:** ^1^ Pulmonology Department University Clinical Hospital of Santiago Santiago de Compostela Spain; ^2^ Interdisciplinary Research Group in Pulmonology Health Research Institute of Santiago de Compostela (IDIS) Santiago de Compostela Spain; ^3^ Pharmacy Department University Clinical Hospital of Santiago Santiago de Compostela Spain; ^4^ University of Santiago de Compostela Santiago de Compostela Spain

**Keywords:** chronic bronchial infection unrelated to PA, inhaled ceftazidime, non‐cystic fibrosis bronchiectasis

## Abstract

**Introduction:**

Inhaled antibiotics reduce the frequency of exacerbations. The objective was to assess the efficacy of inhaled ceftazidime in patients with non‐cystic fibrosis bronchiectasis (NCFB) and concomitant chronic bronchial infection (CBI) caused by potentially pathogenic microorganisms (PPM) other than *Pseudomonas aeruginosa* (PA).

**Material and Method:**

Quasi‐experimental study in 21 patients with exacerbations who developed CBI by a PPM other than PA.

**Results:**

Bacterial infection was resolved in 85.7% patients. Rehospitalizations, length of hospital stay, moderate exacerbations and blood levels of CRP decreased significantly. In addition, SGRQ questionnaire also decreased more than 4 points in 57.1% of the patients.

**Conclusion:**

The results suggest that inhaled ceftazidime in NCFB unrelated to PA is a plausible alternative to the standard therapies used in clinical practice.

## Graphical abstract

Inhaled ceftazidime in patients with non‐cystic fibrosis bronchiectasis and concomitant chronic bronchial infection caused by potentially pathogenic microorganisms other than *Pseudomonas aeruginosa* reduce bacterial infection, rehospitalizations, length of hospital stay, moderate exacerbations, and blood levels of CRP decreased significantly. In addition, SGRQ questionnaire also decreased more than 4 points in 57.1% of the patients.

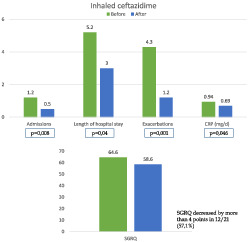

**ABBREVIATIONS**
BQ: bronchiectasis
CBI: chronic bronchial infection
CF: cystic fibrosis
NCFB: non‐cystic fibrosis bronchiectasis
PA: *Pseudomonas aeruginosa*
PPM: potentially pathogenic microorganisms
SGRQ: Saint George Respiratory questionnaire

## 1 INTRODUCTION

In patients with bronchiectasis (BE) unrelated to cystic fibrosis (CF), concomitant chronic bronchial infection (CBI) increases the risk for exacerbations and rehospitalizations, negatively affects quality of life, and increases mortality.^1^ In these cases, inhaled antibiotics reduce bacterial load along with the frequency of exacerbations, with patients showing good tolerance and low rates of resistance. Their effectiveness for CBI by *Pseudomonas aeruginosa* (PA) has been extensively demonstrated.^2^ However, in patients with non‐cystic fibrosis bronchiectasis (NCFB) and concomitant CBI caused by potentially pathogenic microorganisms (PPM) other than PA, evidence about the use of gentamicin^3^ is scarce, and very limited in the case of ceftazidime.^4^

The primary objective of this study was to assess the efficacy of inhaled ceftazidime in patients with NCFB and concomitant CBI caused by PPM other than PA. The secondary objective was to assess the safety of this agent.

## 2 METHODS

A quasi‐experimental study, with comparison of variables before and after the intervention. The status of patients with NCFB and concomitant CBI by a PPM other than PA responsive to this antibiotic was assessed 12 months before and after receiving inhaled ceftazidime. The definitions for BE, CBI, bacterial eradication, and exacerbation were established according to Spanish guidelines.^5^ Bronchiectasis severity was established based on “Bronchiectasis Severity Index”^6^ and E‐FACED.^7^

The sample was composed of adult patients ≥18 years with a history of BE exacerbations (≥2 moderate or ≥1 that required hospitalization) who developed CBI by a PPM other than PA sensitive to ceftazidime in the antibiogram, but not to aminoglycosides. Informed consent was obtained from all participants. We excluded patients who had received inhaled antibiotic therapy within the previous 4 weeks and exhibited a FEV_1_ decrease ≥15% after undergoing a tolerance test to the agent. Inhaled ceftazidime 500 mg was administered twice daily through a mouthpiece using the nebulizer Pari LC Plus or eFlow rapid.

Efficacy endpoints included rehospitalizations, length of hospital stay, exacerbations, dyspnea (based on modified Medical Research Council Scale), and quality of life (based on Saint George Respiratory Questionnaire, SGRQ). Safety endpoints included the occurrence of side effects following treatment initiation that were absent at baseline. The study was approved by the hospital Ethics Committee (2022/24).

### 2.1 Statistical analysis

Data are expressed as mean ± SD. Pre–post intervention differences were assessed using Wilcoxon test. Statistical significance was established at 5%.

## 3 RESULTS

A total of 21 patients received inhaled ceftazidime for 12 ± 14.6 months (range 2–72). The most relevant findings are shown in Table 1. Mean age was 65.5 ± 17 years, 57.1% were women. Bronchiectasis severity was moderate‐to‐severe (mean E‐FACED and BSI were 4 ± 2.5 and 11.1 ± 3.2, respectively). Bacterial infection was resolved in 18/21 patients (85.7%). Six months after treatment withdrawal, four patients (22.2%) experienced a CBI relapse caused by the same pathogen, whereas only a sputum culture was positive for the same microorganism in five (27.8%) patients. Following treatment withdrawal, sputum cultures were negative in nine patients (50%). Rehospitalizations, length of hospital stay, and moderate exacerbations decreased significantly (*p* = 0.008, *p* = 0.04, and *p* = 0.001, respectively; Table 1). A significant reduction was also observed in CRP in blood after the administration of ceftazidime (*p* = 0.046; Table 1). In contrast, dyspnea or lung function parameters did not improve significantly (determined in only 13 patients [61.9%] after ceftazidime inhalation). SGRQ decreased by more than 4 points (minimal clinically relevant difference) in 12/21 patients (57.1%; Table 1).

**TABLE 1:** Characteristics of the patients before and after treatment with inhaled ceftazidime

	Age (years)	Sex	BSI	E‐FACED	CBI for:	Eradication	Baseline admissions	Post‐intervention admissions	Baseline LOS	Post‐intervention LOS	Baseline exacerbations	Post‐intervention exacerbations	Baseline CRP (mg/dl)	Post‐intervention CRP (mg/dl)	Baseline dyspnea (mMRC)	Post‐intervention dyspnea (mMRC)	Baseline FVC (%)	Post‐intervention FVC (%)	Baseline FEV_1_ (%)	Post‐intervention FEV_1_(%)	Baseline SGRQ	Post‐intervention SGRQ
1	57	M	6	0	MSSA	Y	0	0	0	0	3	0	0.22	0.13	2	2	93	111	83	95	56.2	60.1
2	77	W	14	3	HI	Y	0	0	0	0	4	2	2.03	1.88	2	2	81	96	49	59	64.5	59.5
3	74	W	15	6	PM	Y	0	1	0	15	4	1	0.59	0.52	3	3	86		43		96.1	97.7
4	53	M	12	5	BB	Y	6	1	12	6	5	1	1.15	0.36	2	2	43.1		33.4		90.3	90.3
5	76	W	12	4	PM	Y	1	0	3	0	4	0	0.62	0.57	1	1	112		83		90.6	66.7
6	55	W	7	1	EC	Y	0	0	0	0	4	2	0.21	0.05	1	1	76.5	79	58	63	70.8	72.7
7	78	M	13	5	HI	Y	1	0	5	0	4	1	2.37	1.80	1	1	103	87.9	81	72.4	58.9	53.1
8	59	W	9	3	PM	Y	1	0	6	0	2	0	0.05	0.24	1	1	109		91		56.3	51.9
9	43	W	9	1	EC	Y	0	0	0	0	8	0	0.46	0.77	1	1	137.3	126	121.2	112	48	50.1
10	68	W	10	3	EC	Y	2	0	14	0	1	1	1.15	0.46	1	1	94	91	70	71	26	6.8
11	70	M	15	8	StP	Y	1	0	16	0	10	8	1.78	1.39	3	3	46		31		86.3	90.6
12	78	M	9	3	EC	Y	0	0	0	0	4	0	0.84	0.39	1	1	103	111	67	64	22.2	38.7
13	60	W	5	0	AX	Y	0	0	0	0	4	0	0.19	0.31	1	1	112	123	83	89	58.9	41.2
14	83	W	10	4	PM	Y	0	0	0	0	4	1	0.86	0.22	3	2	72		83		88	83.6
15	85	M	11	4	PM	Y	0	0	0	0	4	1	2.07	0.12	3	3	52		58		71.1	71.8
16	75	M	15	8	EC	Y	2	1	10	8	8	4	1.11	1.19	3	3	49	60	41	35	97.7	93.1
17	79	M	9	3	HA	No	0	0	0	0	4	0	0.35	0.37	0	0	59		56		21.2	9.6
18	18	W	8	2	HI	Y	2	0	3	0	4	1	0.89	0.88	0	0	113.9	100	102.2	93	51.1	18
19	79	M	15	8	AX	No	3	2	12	9	4	2	1.12	0.97	3	3	47.6	49	34.7	33	97.7	96.1
20	73	W	15	8	PM	Y	3	3	14	10	4	0	0.94	0.96	3	3	62.2	71	40.3	45	56.9	51.2
21	37	W	13	5	EC	No	4	3	14	14	1	1	0.69	0.91	1	1	70	82.7	49	57.8	47.6	26.9
X ± SD	65.5 ± 17		11.05 ± 3.19	4 ± 2.5			1.2 ± 1.6	0.5 ± 1	5.2 ± 6.1	3 ± 5.1	4.3 ± 2.1	1.2 ± 1.8	0.94 ± 0.66	0.69 ± 0.53	1.7 ± 1.1	1.7 ± 1	82 ± 27.3	91.4 ± 23.3	64.7 ± 24.7	68.4 ± 23.9	64.6 ± 24.9	58.6 ± 28.5
							0.008		0.04		0.001		0.046		0.317		0.382		0.727			

Abbreviations: AX, *Achromobacter xylosidans*; BB, *Bordetella bronchiseptica*; BSI, bronchiectasis severity index; CBI, chronic bronchial infection; CRP, C‐reactive protein; EC, *Escherichia coli*; FEV_1_, forced expiratory volume during the first second; FVC, forced vital capacity; HA, *Hafnia alvei*; HI, *Haemophilus influenzae*; LOS, length of hospital stay; M, man; mMRC, modified *Medical Research Council scale*; PT, *Proteus mirabilis*; MSSA, methicillin‐sensitive *Staphylococcus aureus*; SGRQ, *Saint George Respiratory Questionnaire*; StP, *Streptococcus pneumoniae*; W, woman; Y, yes.

In relation to side effects, a patient developed cough and three episodes of dyspnea with mild wheezing that led to treatment withdrawal 6–7 months after initiation.

## 4 DISCUSSION

Our results demonstrate that inhaled ceftazidime achieves bacterial eradication, reduces exacerbations (severe and moderate), decreases systemic inflammation, and improves quality of life in a significant proportion of patients with NCFB and CBI unrelated to PA.

To the best of our knowledge, this is the first study to assess the impact of inhaled ceftazidime in a group of selected patients. Although antibiotic therapy has been proven to be effective in the management of CBI secondary to PA infection, evidence on its effectiveness for CBI caused by other pathogens is limited. Studies conducted to assess the efficacy of an inhaled antibiotic in patients with BE and concomitant CBI should consider the bacterial eradications achieved and the reduction of exacerbations, since they are associated with quality of life deterioration and a higher risk of rehospitalization and mortality.^8^

Ceftazidime is rarely used in BE related to CF. In a cohort of 11 patients, a significant improvement in lung function was only achieved during a period of 3 months, but the treatment was not effective in reducing the duration of intravenous antibiotic therapy. In addition, the evolution of exacerbations was not assessed.^4^ In NCFB and CBI secondary to PA infection, rehospitalizations (0.6 vs. 2.5 [*p* = 0.023]) and length of hospital stay (13.1 vs. 57.9 days [*p* = 0.033]) decreased in the group who received inhaled tobramycin and ceftazidime, but without any improvement in lung function.^9^ However, since it is a combination therapy, it is difficult to determine the agent that exerted these beneficial effects. In a study involving 14 patients with NCFB and concomitant CBI unrelated to PA, inhaled gentamicin was effective in achieving bacterial eradication in 13 patients (92.8%).^3^ Inhaled gentamicin, reduced exacerbations, increased time to first exacerbation, and improved quality of life in patients with BCI due to PPMs, as compared with placebo. Conversely, no improvements were observed in lung function or C‐reactive protein. In another study in patients with NCFB and CBI by PA or other PPM, inhaled ciprofloxacin reduced significantly sputum bacterial load, and bacterial eradication was achieved in a higher number of patients upon treatment completion, as compared to placebo, with patients showing good tolerance.^10^ Of note, a sub‐analysis of the results obtained was not performed in any of the two studies,^3^, ^10^ only in patients with CBI secondary to PPM other than PA. Therefore, it is difficult to determine the efficacy of antibiotics in the latter.

The study has some limitations that may influence in the results: The sample is small; pre‐intervention data collection was retrospective; comparison between days and number of admissions must be interpreted with caution, because they are collected in a different way; lastly, the study was conducted in a single center without validation series of the results.

In conclusion, our results are consistent with the literature in terms of percentage of eradications attained (87.5% vs. 92.8%^3^), reduction of exacerbations (4.3 ± 2.1/1.2 ± 1.8 exacerbations vs. 0 with gentamicin/1.5 with placebo^3^), of rehospitalizations (1.2 ± 1.6/0.5 ± 1 admissions vs. 0.6 with tobramycin + ceftazidime/2.5 with symptomatic treatment^9^), and of length of stay (5.2 ± 6.1/3 ± 5.1 days vs. 13.1 days with tobramycin + ceftazidime/57.9 days with symptomatic treatment^9^). Lung function did not improve either in our study or in previous studies.^3^ Patients show relatively good tolerance, with limited adverse effects.

The results obtained in this study suggest that inhaled ceftazidime in NCFB unrelated to *Pseudomonas aeruginosa* is a plausible alternative to the standard therapies used in clinical practice. Large, long prospective studies are necessary to confirm the promising results obtained in this study.

## CONFLICT OF INTEREST

Authors declare no conflict of interest.

## ETHICS STATEMENT

The study was conducted in accordance with the principles of the Declaration of Helsinki and Good Clinical Practice guidelines. The study was approved by the hospital Ethics Committee (2022/24).

## AUTHOR CONTRIBUTIONS

Vanessa Riveiro conceived and designed the study, performed data analysis and interpretation, reviewed intellectual content, and approved final manuscript. Ana Casal performed data analysis and interpretation, conducted a critical review of intellectual content, and approved final manuscript. José M. Álvarez‐Dobaño performed data analysis and interpretation, conducted a critical review of intellectual content, and approved final manuscript. Tamara Lourido performed statistical analysis, performed data analysis and interpretation, conducted a critical review of intellectual content, and approved final manuscript. Pedro Suárez‐Artime performed data analysis and interpretation, conducted a critical review of intellectual content, and approved final manuscript. Carlota Rodríguez‐García performed data analysis and interpretation, conducted a critical review of intellectual content, and approved final manuscript. Lucía Ferreiro performed data analysis and interpretation, conducted a critical review of intellectual content, and approved final manuscript. María E. Toubes performed data analysis and interpretation, conducted a critical review of intellectual content, and approved final manuscript. Luis Valdés conceived and designed the study, performed data analysis and interpretation, reviewed intellectual content, and approved final manuscript.

## Data Availability

The data that support the findings of this study are available on request from the corresponding author. The data are not publicly available due to privacy or ethical restrictions.
